# Human Activity Changes During COVID‐19 Lockdown in China—A View From Nighttime Light

**DOI:** 10.1029/2021GH000555

**Published:** 2022-08-01

**Authors:** Xuejun Wang, Guangjian Yan, Xihan Mu, Donghui Xie, Jiachen Xu, Zhiyu Zhang, Dingdan Zhang

**Affiliations:** ^1^ State Key Laboratory of Remote Sensing Science Jointly Sponsored by Beijing Normal University and Aerospace Information Research Institute Chinese Academy of Sciences Beijing China; ^2^ Beijing Engineering Research Center for Global Land Remote Sensing Products Faculty of Geographical Science Beijing Normal University Beijing China

## Abstract

Strict lockdowns were implemented in China to fight Coronavirus Disease 2019 (COVID‐19). We explored the nighttime light (NTL) of China's four cities in five stages of COVID‐19 including case free period, newly appeared period, rising period, outbreak period, and stationary period. Using six categories of points of interest data (“company,” “recreation,” “healthcare,” “residence,” “shopping,” and “traffic facility”) and random forest models, we found that dimming light of four cities is associated with the epidemic development and human activity changes. When confirmed cases appeared, healthcare associated NTL radiance increased rapidly in Wuhan and Guangzhou, but decreased in the fourth and fifth stages. Companies in all cities were resuscitated in the fifth stage, while companies in Guangzhou was resuscitated in the fourth stage. Shopping related NTL radiance in Wuhan increased quickly in the fifth stage which indicated some resuscitation. In addition, compared to gross domestic product, the trend in electric power consumption was consistent with the trend in NTL radiance. The above findings contribute to the making of control policies for COVID‐19 as well as other infectious diseases.

## Key Points

- Dimming light during Coronavirus disease 2019 lockdown observed by satellite coincides with the stage of epidemic development
- Changes in six categories of human activity can be considerably explained by nighttime light (NTL) changes (around 52%)
- GDP (or electric power consumption) has a consistent trend with the NTL radiance

## 1 Introduction

The human‐to‐human transmission of the COVID‐19 was first reported in Wuhan, China, in early 2020 (Li et al., 2020; Sohrabi et al., 2020; Tian, Hu, et al., 2020; Wang, Horby, et al., 2020; Zu et al., 2020). The disease has spread globally and the cumulative confirmed cases exceeded 200 million, including more than 4 million deaths (https://covid19.who.int/, as of 1 September 2021). The severe pandemic has had a growing negative impact globally and implementing effective measures to control the spread of the virus is still essential.

In response to this pandemic, lockdown restrictions were first applied to Wuhan, subsequently covering the whole of China (Su et al., 2020). Many people isolated themselves and human habits and activities changed dramatically, affecting their work and living environment. Lockdown measures can reduce the rate and limit the spread of the COVID‐19, which have been proved effective in mitigating the pandemic (Chen, Zhang, et al., 2020; Chinazzi et al., 2020; Musinguzi & Asamoah, 2020). However, lockdowns also correspond to a drastic freeze in population flows (Chen, Zhang, et al., 2020) which resulted in economic setbacks and social problems such as increasing poverty, inequality, and domestic abuse (Bonaccorsi et al., 2020; Nicola et al., 2020; Straka et al., 2020). The outbreak in China has been better controlled in the summer of 2020, but confirmed cases have been rising globally. In light of this, analyzing the impact of COVID‐19 on people's activities and response to policies related to the lockdown is urgent for China to refine its pandemic control measures and has significant implications for other countries.

Changes in human activity before and after lockdown, and the consequent impacts, can be monitored through satellites. Nighttime light (NTL) has been proven to be an effective indicator of socio‐economic parameters (Bennett & Smith, 2017; Chen et al., 2021; Dou et al., 2017; Levin & Zhang, 2017; Levin et al., 2020; Ma et al., 2014). Low‐light night satellite sensors can detect dimming and loss of lighting after disasters (Kohiyama et al., 2010; Roman et al., 2019) and wars (Jiang et al., 2017; Li et al., 2015, 2017), and brightening and increasing of lighting because of urbanization (Dou et al., 2017; Shi et al., 2014) and fire (Polivka et al., 2016). Visible Infrared Imaging spectroRadiometer Suite (VIIRS) instrument's Day/Night Band sensor, onboard the Suomi National Polar‐orbiting Partnership and joint polar satellite system satellite platforms, is the main source of nighttime light (NTL) data, which has significantly improved the sensitivity of light capture, the range of light recording values and the spatial resolution compared to Defense Meteorological Satellite Program–Operational Linescan System, providing a unique perspective and a stable and convenient way to study human activities (Bennett & Smith, 2017; Chen et al., 2019; Elvidge et al., 2013). Nightlight data can thus reflect and monitor the response of citizens to the lockdown and segregation policies (Liu et al., 2020). Furthermore, points of interest (POIs) data featuring geographic and category information can characterize human activities (Chen et al., 2019). The combination of POIs data and night light data allows for a more comprehensive understanding of the effects of lockdown on human behavior changes caused by COVID‐19.

The severity of the epidemic among cities and stages of the pandemic development can affect NTL radiance, and the degree of radiance change in areas of human activity on NTL images during the outbreak also varies by category. However, the relationships between the COVID‐19 epidemic and different categories of human activities lacked quantitative evaluation. Additionally, specific responses in terms of socio‐economic parameters related to human activities in China cities remain unclear. In this study, we obtained NTL images, POIs data, gross domestic product (GDP) data, and electric power consumption (EPC) data. Using four case study cities, we estimated the correlations between human activities and NTL radiance and evaluated contributions of each human activity category to the NTL change with the random forest method. Consequently, we obtained the responses of different human activities to the lockdowns in four cities. We also examined the changes in GDP and EPC for the first quarter of the last 5 years and obtained the impact of the COVID‐19 lockdowns on socio‐economic variables from NTL images.

## 2 Data and Methods

### 2.1 Study Area and Period Division

This study focused on four cities including Beijing, Wuhan, Shanghai, and Guangzhou. Unnatural landscape areas and permanent residents in Shanghai are the largest, followed by Beijing, Guangzhou, and Wuhan (Table S1 in Supporting Information S1). Guangzhou also has many biomedical companies and well‐developed manufacturing industries (Jiang, 2014), and foreign trade industries. All cities are major metropolitans in China with varying degrees of epidemics. We obtained total confirmed cases data from 1 January to 31 March in China from the data set of the COVID‐19 epidemic (https://github.com/Estelle0217/COVID‐19‐Epidemic‐Dataset), details are shown in Table S1 in Supporting Information S1.

**Figure 1:**
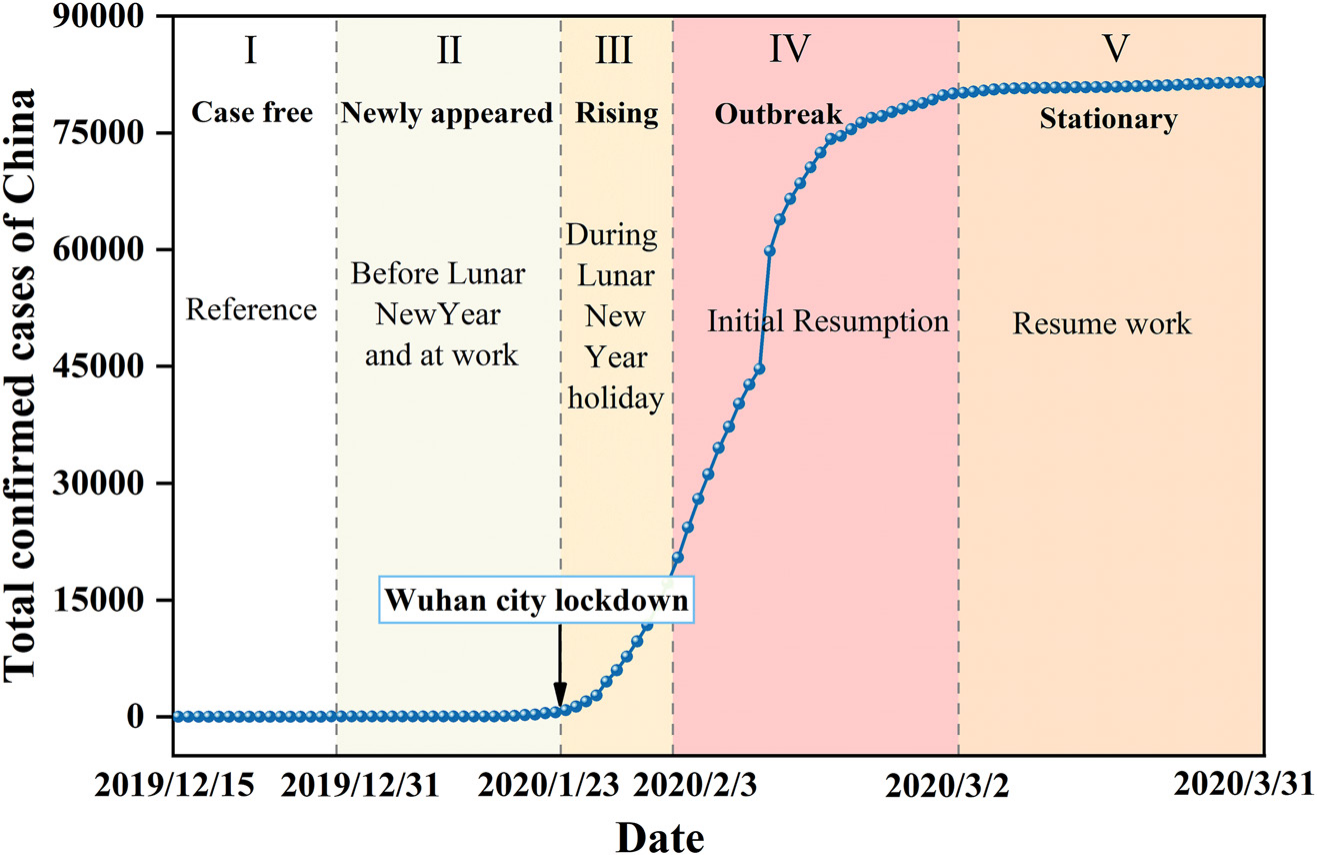
Division of Coronavirus Disease 2019 trend in China. The blue dotted line represents the total confirmed cases in China every day. Different background colors indicate different stages.

We divided the COVID‐19 trend into five stages based on the growth rate of the total confirmed cases and the Lunar New year holiday, that is, case free period (stage I, 15–31 December 2019), newly appeared period (stage II), rising period (stage III), outbreak period (stage IV), and stationary period (stage V). And stage I was used as the reference period. The time nodes and description are also shown in Figure 1. Before 16 January, confirmed cases were only reported in Wuhan. After that time, cases occurred in other cities (Chen, Zhang, et al., 2020). And total confirmed cases of China increased rapidly after 22 January. On 23 January, strict lockdown restrictions were applied to Wuhan, and covered the whole country within a few days (Su et al., 2020). Then, the growth rate reached its highest in stage IV. By stage V, the growth rate has moderated and the total number of diagnoses is approximately 81 thousand. Focusing specifically on the four cities, the trend of their epidemic is basically consistent with that of the whole country, with a particularly serious one in Wuhan, where more than 47,000 cases have been confirmed and up to 50,000 in stage V. Meanwhile, data of the same period from 2016 to 2019 were also divided into five stages for comparison with 2020, and time divisions in each year are shown in Table S2 in Supporting Information S1.

### 2.2 Data Sets

#### 2.2.1 VIIRS Data VNP46A2

VIIRS/NPP Gap‐Filled Lunar BRDF‐Adjusted Nighttime Lights Daily L3 Global Linear Lat Lon Grid (VNP46A2) data belonging to NASA Black Marble product is a daily, moonlight‐ and atmosphere‐corrected nighttime radiance product with a resolution of 500 m, and available from 2012 onward (Román et al., 2018). This product contains seven Science Data Sets layers in which NTL radiance and quality flags were used in this study. To estimate changes in urban NTL radiance during the COVID‐19 epidemic, data of 1 January to 31 March (Q1) from 2016 to 2020 and 15–31 December from 2015 to 2019 were downloaded from the website of LAADS DAAC (https://ladsweb.modaps.eosdis.nasa.gov/). All NTL data were processed to synthesize images for each stage in Figure 1, details of which can be found in Text S1 in Supporting Information S1. Data of 15–31 December 2019 were synthesized as reference images (stage I) because this period was the closest to the start of the COVID‐19. In addition, it can be assumed that background values of the NTL radiance did not change compared to the first quarter of 2020, and the change in NTL radiance is caused by changes in human activity under the influence of the epidemic.

#### 2.2.2 POIs Data

**Figure 2:**
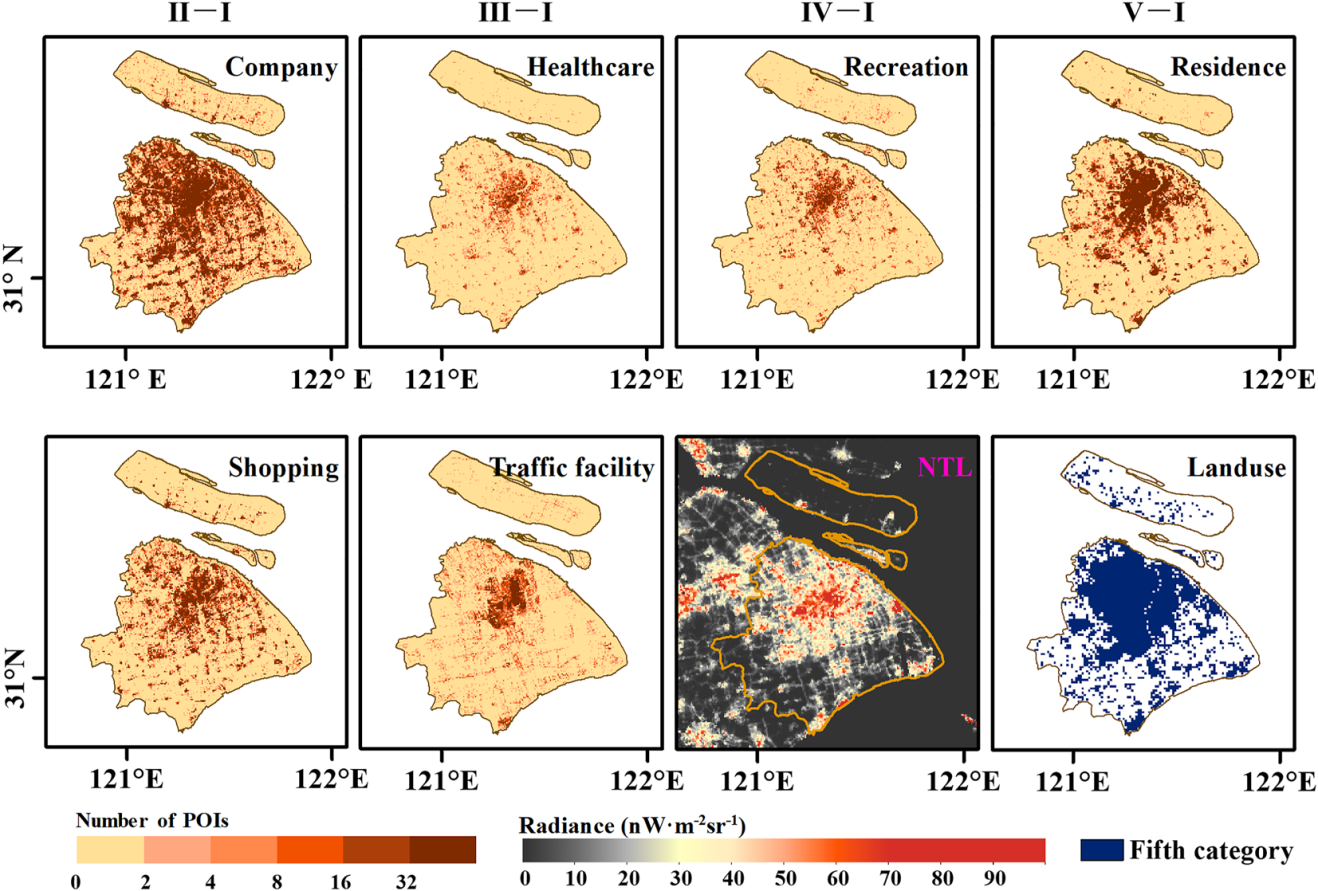
Map of points of interests for company, healthcare, recreation, residence, shopping, traffic facility, and corresponding nighttime light radiance image of stage I in 2020, and unnatural landscape area (the fifth category) of Shanghai.

POIs data contain a large number of different classes of points with name, address, and location. These points reflect various human activities (Chen et al., 2019). We crawled six human activity categories of POIs data in 2020 of four cities from Baidu Map (https://map.baidu.com/), including company, healthcare, recreation, residence, shopping and traffic facility, which are classified by the first‐level industry. Each category contains tens of thousands to hundreds of thousands of points (Table S3 in Supporting Information S1). All these data types are related to human activity intensity and can be further overlapped in NTL images. The point density of each category (Figure 2) was calculated and used as a proxy for human activity to model the relationship with NTL.

#### 2.2.3 Land Use Data

Land use remote sensing data in 2018 of four cities were downloaded from Resource and Environment Science and Data Center (http://www.resdc.cn/Default.aspx). This product divides the world's land surface into seven categories, and the fifth category (urban and rural, industrial and mining, and residential land) of four cities was used. The development of urbanization and the enhancement of human activities have gradually replaced the original natural landscape. Therefore, concrete floors and tall buildings have become representative of the urban landscape, and NTL are mainly distributed in these places. We extracted unnatural landscape areas (the fifth category, Figure 2) to mask the NTL images.

#### 2.2.4 GDP and EPC Data

Q1 GDP data and EPC data from 2016 to 2020 are from the National Bureau of Statistics and local statistical bureaus (https://data.stats.gov.cn/english/easyquery.htm?cn=E0102), of which Q1 EPC data from 2016 to 2018 in Beijing is missing. These data were used to analyze the relationship between the NTL changes and socioeconomic changes during the epidemic.

### 2.3 Analysis of NTL Radiance Change

The average NTL radiance images were calculated in each stage from 2016 to 2020 in different cities. To evaluate the impact of the epidemic on the change in NTL radiance, we performed the analysis by the following two components: (a) The differences between the NTL images of stage II to V and those of stage I in 2020 were calculated to obtain the NTL radiance change since the beginning of the epidemic. Meanwhile, the differences between stage II to V and stage I in 2019, which reflect the NTL radiance change without the epidemic, were also calculated for comparison with that in 2020. (b) In order to avoid the randomness of the NTL radiance change obtained by the data of 2019, yearly changes of NTL radiance in unnatural landscape areas of each stage in 5 years were calculated.

### 2.4 Anthropogenic Contributions to NTL Change at Each Stage

The RF algorithm is an ensemble learning algorithm aggregating multiple classification and regression trees, each of which is assigned by an independently sampled random vector (Breiman, 2001). Training samples are selected by bootstrap iterations from the original samples, and for each new training sample, a decision tree is generated using a random feature selection method. The final output value is obtained by averaging the predictions of all decision trees. The RF algorithm uses out‐of‐bag (OOB) data to estimate the internal error since about 37% of samples in each tree are not selected when bootstrap training samples are randomly selected from the original sample set each time (Kuhn, 2008; Liaw & Wiener, 2002).

Considering the stability and accuracy, three parameters in the random forest were tuned: (a) the number of decision trees (from 100 to 500 at intervals of 25); (b) the number of random features used at each node (from 2 to 6 at intervals of 1); and (c) the minimum number of leaf nodes (from 1 to 10 at intervals of 2). And the application of RF to analyze the anthropogenic contributions to NTL change at different stages of COVID‐19 is given below. The number of POIs at pixel level (500 × 500 m) in the six categories (“company”, “recreation”, “healthcare”, “residence”, “shopping”, “traffic facility”) was used as the independent variable, and the NTL radiance of each stage in 2020 was used as the dependent variable. The root mean square error (RMSE) and the coefficient of determination (R^2^) of the OOB data were used to evaluate the model (Wright & Ziegler, 2017). By interpreting the RF models, a feature contribution method (Palczewska et al., 2013) was used. For each of the six categories, the contribution of the sample to the target value was calculated, and then medians of feature contributions as a proxy for the “standard level” of variable contributions were obtained with higher absolute values indicating a larger contribution of the category to the NTL radiance.

## 3 Results

### 3.1 NTL Radiance Changes From 2016 to 2020

**Figure 3:**
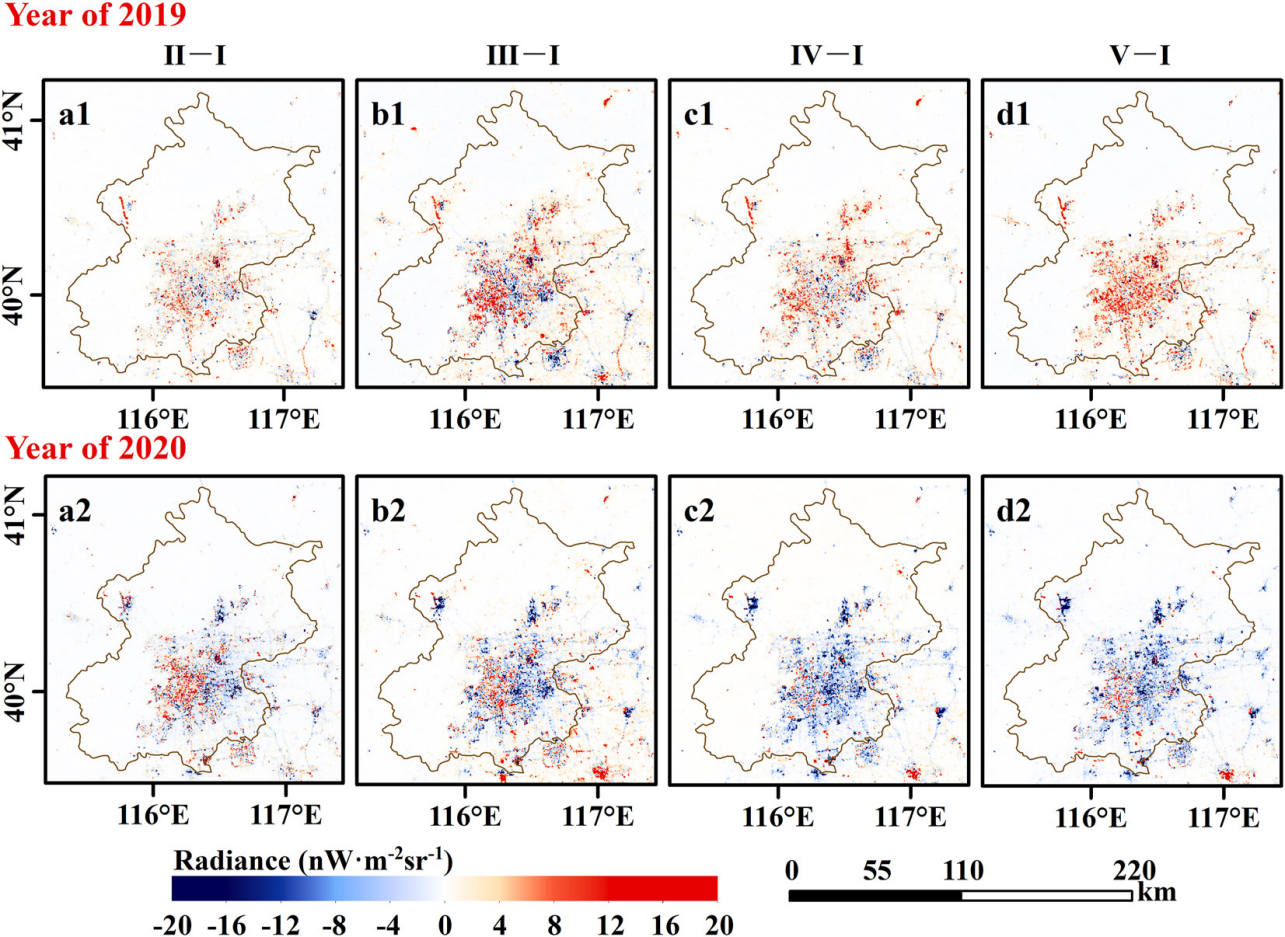
Nighttime light (NTL) radiance changes in Coronavirus Disease 2019 development stages of Beijing. The upper panel and bottom panel depict the NTL radiance change in 2019 and 2020, respectively. The columns from left to right are NTL radiance difference (a) between stage II and stage I, (b) between stage III and stage I, (c) between stage IV and stage I, and (d) between stage V and stage I. Compared to the NTL radiance in 2019, the white part indicates little or no NTL change, the red part indicates brightening, and the blue part indicates dimming.

**Figure 4:**
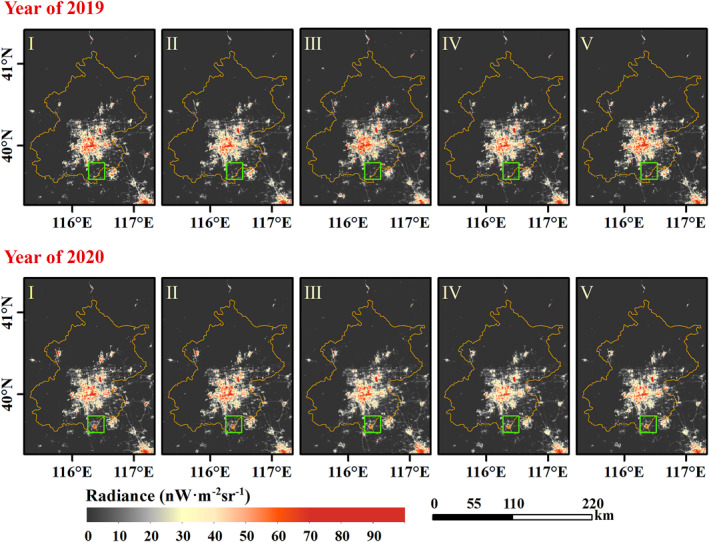
Average Nighttime light (NTL) radiance distribution of Beijing in different stages. The upper panel depicts the NTL radiance in 2019 and the bottom panel depicts the NTL radiance in 2020.

Contrary to the increasing total confirmed cases, the NTL dimming compared to stage I is detected from the nighttime satellite images in 2020 with the COVID‐19 outbreak (Figure 3, and Figures S2 to S4 in Supporting Information S1). As shown in these figures, the white part indicates little or no NTL change compared to stage I, the red part indicates brightening, and the blue part indicates dimming. Except for the unchanged area, the blue part in 2020 dominates a larger area than in the previous year, and NTL dimming is the most noticeable in stage IV shown in Figure 3 (c2). Besides, compared to the NTL images in 2019 of Beijing, Wuhan, and Shanghai and 2018 of Guangzhou, prominent NTL brightening is exhibited in Figure 4, and Figures S5 to S7 in Supporting Information S1, such as the airport in Beijing (example: Beijing Daxing International Airport, regions in the green rectangle of Figure 4) and road in Wuhan (example: Mulan Avenue, regions in the green rectangle of Figure S4 in Supporting Information S1).

**Figure 5:**
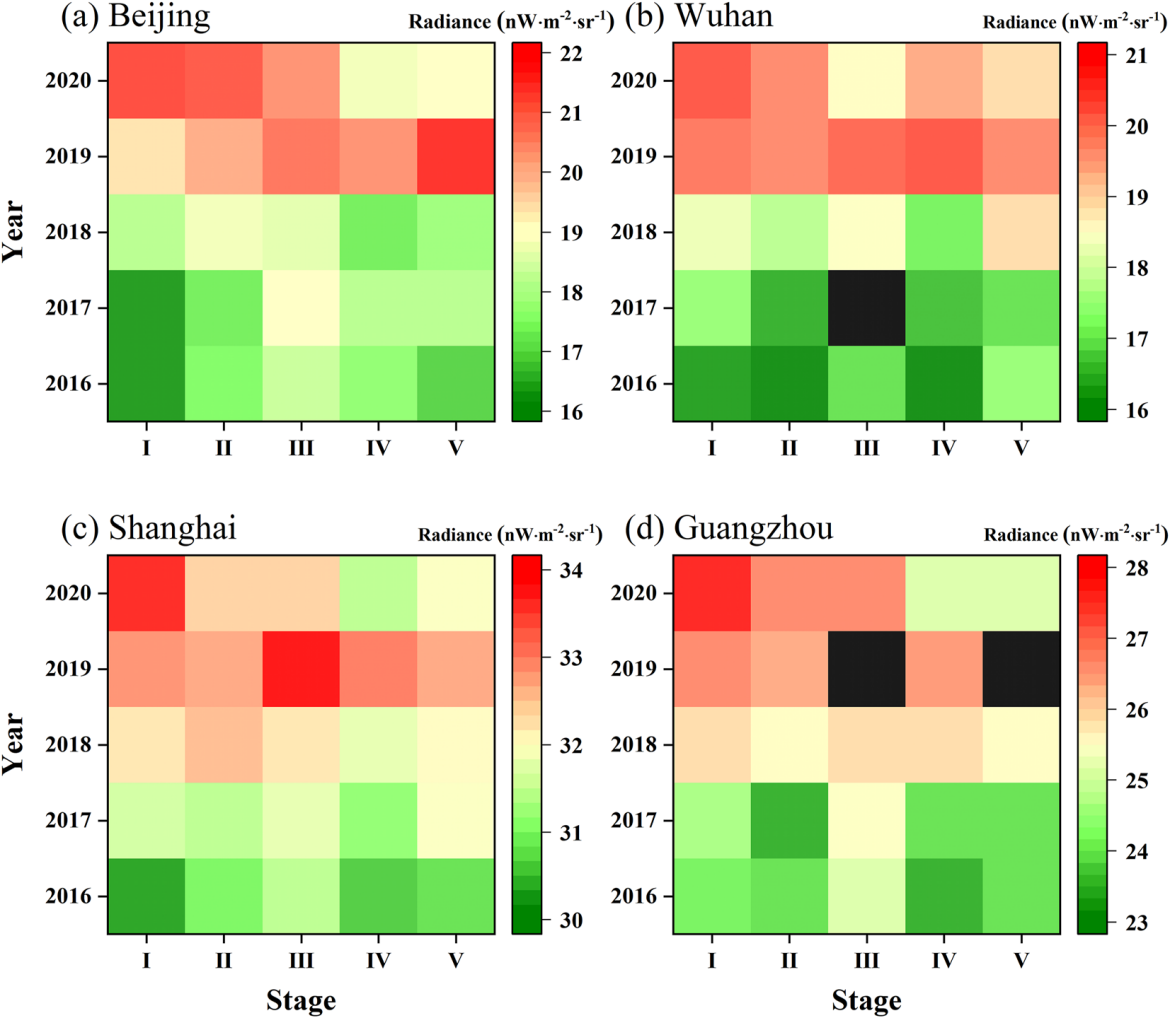
Yearly changes of Nighttime light radiance of five stages for the four cities: (a) Beijing, (b) Wuhan, (c) Shanghai, and (d) Guangzhou. The black blocks in (d) represent no data.

To examine whether the dimming is unique in 2020, the average NTL radiance in unnatural landscape areas of each stage in different years is calculated. The overall NTL radiance is increasing from 2016 to 2019, with peaking in 2019 (Figure 5). As illustrated in Figure 5, NTL radiance shows a greater divergence at different stages. The NTL of the four cities in stage I is mainly brightening over time, but the other four stages are visibly dimmed in 2020. Due to the gradual stabilization of the epidemic, NTL radiance recovered subsequently after stage IV to some extent in Beijing, Shanghai, and Guangzhou, but still lower than stage I (reference images). And for Wuhan, the lowest NTL radiance of 2020 is in stage III, showing a tendency to rebound in stage IV, which is relevant to the assistance given to Wuhan from all over the world and the construction of temporary hospitals (Liu et al., 2020; Sohrabi et al., 2020).

### 3.2 Impact of COVID‐19 on Human Activities From NTL Images

We applied the RF model to evaluate how the COVID‐19 lockdown influences human activities as reflected from NTL images at the pixel level. Table 1 shows the results of the model evaluation. The NTL radiance can be partly explained by a combination of six categories of human activity with R^2^ ranging from 0.427 to 0.651 and RMSE about 10 nW · m^−2^sr^−1^. The NTL radiance can be best depicted by POI in Beijing (average R^2^ is 0.633 and RMSE is 7.634 nW · m^−2^sr^−1^) and worst‐fitted in Shanghai (average R^2^ is 0.437 and RMSE is 15.817 nW · m^−2^sr^−1^).

**Table 1:** Assessment of RF Models in Each Stage for Four Cities

Stage	Beijing		Wuhan		Shanghai		Guangzhou	
	R^2^	RMSE	R^2^	RMSE	R^2^	RMSE	R^2^	RMSE
I	0.611	8.418	0.532	8.490	0.432	16.864	0.466	11.837
II	0.626	8.202	0.513	8.481	0.463	14.783	0.472	11.604
III	0.630	7.663	0.516	7.745	0.427	15.610	0.478	11.481
IV	0.651	6.780	0.478	8.906	0.433	14.967	0.486	10.164
V	0.648	7.105	0.524	8.136	0.432	16.86	0.487	10.051

*Note.* The unit of root mean square error (RMSE) is nW · m^−2^sr^−1^, and each column represents information on model evaluation parameters at different stages.

**Figure 6:**
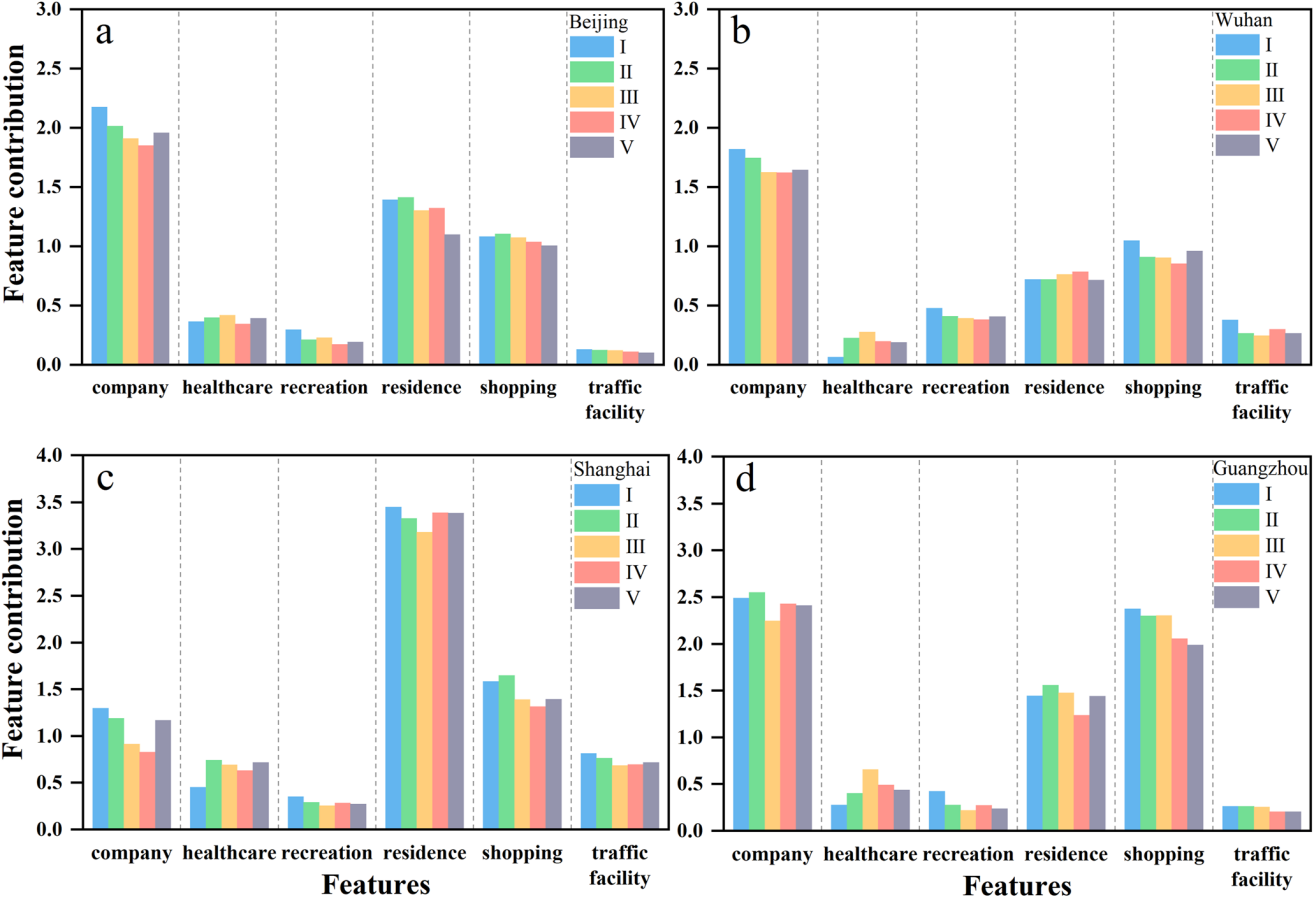
Medians of feature contributions for each human activity category.

We further used medians of feature contributions for representatives to quantitatively analyze the variations in human activity. As shown in Figure 6, there are three significant variables for four cities: company, residence, and shopping, the point density of which are significantly larger than the other three (Figure 2). Except for healthcare and residence, the contributions of the other four categories all declined due to the epidemic, and the contribution of healthcare increased in four cities. The contributions of the company to NTL radiance are the largest among six categories in Beijing, Wuhan, and Guangzhou, but residence makes the largest contribution in Shanghai probably attributable to the large population and the areas of unnatural landscapes (Table S1 in Supporting Information S1). Additionally, the specific performance of the four cities varies over the five stages.

For Beijing (Figure 6a), the contribution of the company gradually declined from stage I to IV and recovered in stage V, which is still lower than stage I. This fluctuation is in agreement with the change of NTL (Figure 5a). The contribution of healthcare increased after cases were reported. In stage III, the contribution of residence has a decrease which may be related to the large population movement before the lockdown (before the Spring Festival), but a slight rebound appears in stage IV. The contributions of shopping and recreation were both reduced on the whole. And the contribution of traffic facility is gradually decreasing.

For Shanghai (Figure 6c), the contribution of the company started to recover in stage V. The contribution of shopping, the second largest among the six variables, also has the same trend. After the confirmed cases appeared, the contribution of residence from stage II to III declined and has a noticeable growth in stages IV and V, which may be resulted by staying at home and increasing family recreational activities. Moreover, traffic facility contributions varied as a "U" shape with the lowest value occurring in stage III.

For Guangzhou (Figure 6d), despite the overall reduction in the company's contribution from stage III to V, the specific pattern of change differs from that in the other three cities. The company started to recover in stage IV. By 31 March 2020, there are 440 confirmed cases, less than in Beijing (580), Shanghai (516), and Wuhan (50,007). Comparatively, the outbreak in Guangzhou is less severe among the four cities and many medical manufacturing companies resumed work early in response to the outbreak. The recovery trend began to emerge in March, with the acceleration of the resumption of work and production (Wang & Su, 2020). Moreover, many companies transformed into the production of medical supplies, rushing to work day and night. In March, an outbreak was occurring abroad while the epidemic entered a stable stage in China. Therefore, the high demand for foreign trade led to more companies' transformation. The contribution of healthcare increased rapidly after cases were reported, and declined a little in stage IV and V. In addition, the contribution of shopping gradually declined from stage II.

Wuhan, in which the earliest and most stringent measures to reduce people's exposure to COVID‐19 were taken, exhibits a different situation compared to the other three cities, especially for residence and traffic facilities (Figure 6b). Given traffic facilities, the contribution has a larger value in stage IV, when many healthcare professionals all over the country were sent (Gan et al., 2020) and large quantities of socially donated emergency supplies continue to be delivered to Wuhan. Compared to the other three cities, the contribution of residence increased when confirmed cases appeared and peaked at stage IV. The contribution of the company declined in stage II to IV and has a little rebound in stage V. Also, shopping has the same trend with company but increased rapidly in stage V that indicate some signs of resuscitation. Healthcare is lowest among six categories in stage I but increased considerably after confirmed cases appeared, but decreased to some extent in stage IV and V.

### 3.3 Impact of COVID‐19 on Socio‐Economic Variables From NTL Images

**Figure 7:**
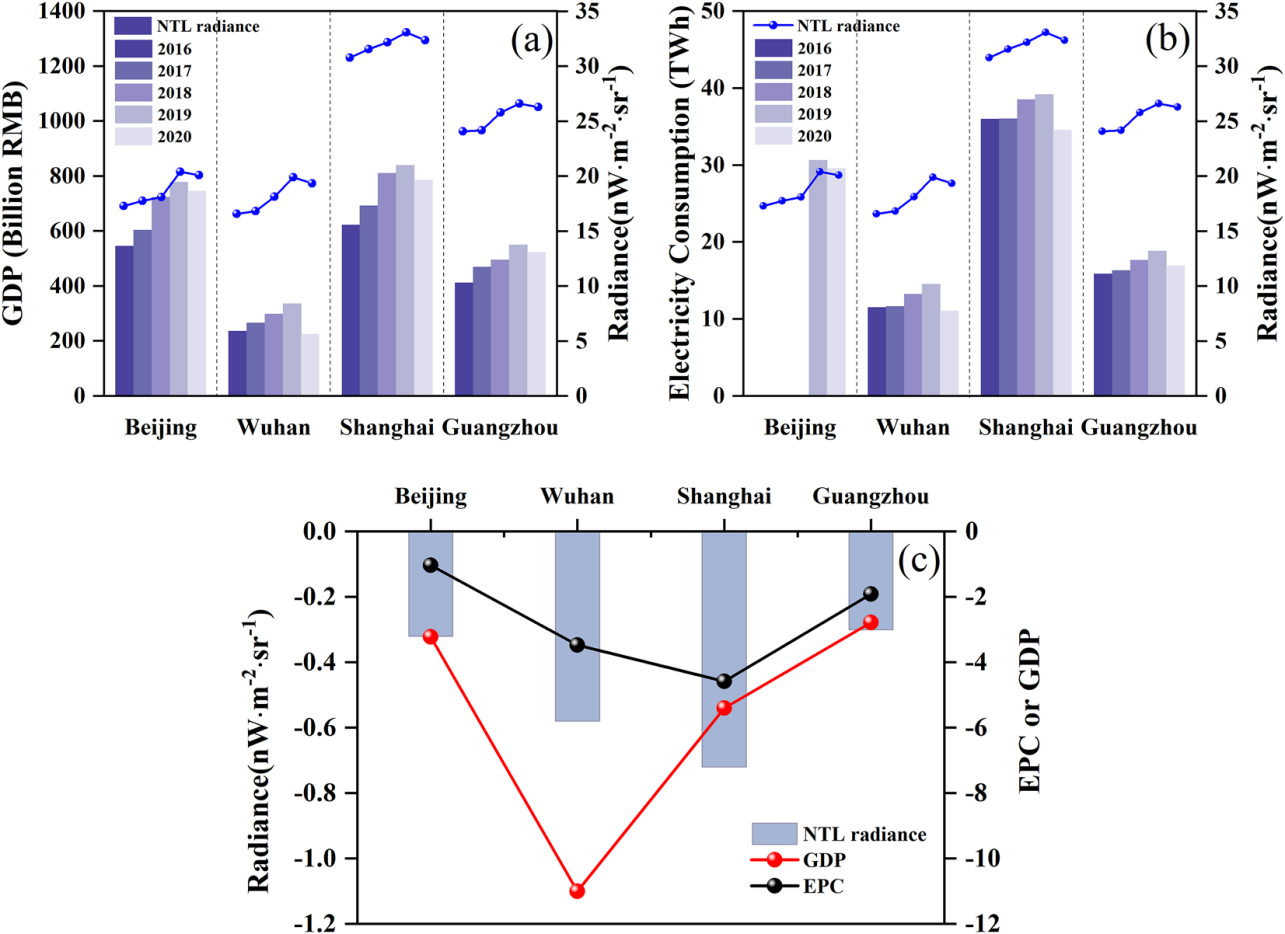
Changes of gross domestic product (a) and electric power consumption (b) with Nighttime light radiance in Q1 (Missing electricity consumption data of Beijing for 2016 and 2017), and differences of these variables between Q1 of 2020 and that of 2019 (c).

The COVID‐19 epidemic not only poses a serious threat to public health but also significantly hampers global economic growth (Bai et al., 2020; Lai et al., 2020). China's electricity demand has long been on the rise with the expansion in construction and economic activity (Liang & Liang, 2017; Lin & Wu, 2017). However, in response to the COVID‐19 outbreak, lockdown measures were applied and socio‐economic issues also emerged. To capture the impact of the COVID‐19 on socio‐economic parameters related to human activities in China cities, we focused on GDP and EPC to analyze the change using NTL radiance. Figure 7 shows the change in NTL, GDP, and EPC of Q1 from 2016 to 2020. The NTL radiance and GDP (or EPC) for the four cities generally show a consistent change trend with a growth trend from 2016 to 2019 and a decrease in 2020.

With the lockdown leading to a decrease in people's activity outside, such as shopping, recreation, and company, GDP and EPC declined in Q1 of 2020 compared to 2019 especially for Wuhan, the worst affected area by the COVID‐19 outbreak. A more detailed analysis is shown in Figure 7c and Table S4 in Supporting Information S1. Compared to the other three cities, Shanghai has the largest decrease in NTL radiance, followed by Wuhan, Beijing, and Guangzhou. Meanwhile, the maximum reduction of EPC is also in Shanghai, not in Wuhan, possible causes are the construction of new hospitals and the smallest electricity consumption in Wuhan compared with the others. In addition, the other three cities experienced a smaller slowdown in GDP compared to Wuhan.

When we compare the details, we note that NTL and EPC reductions are well matched to each other with an R^2^ of 0.92, but the R^2^ of NTL and GDP reductions is 0.36 (Table S4 in Supporting Information S1), probably because the NTL radiance reflects electricity consumption. Furthermore, human activities change dramatically during the epidemic. Contributions of residence are increased in stage IV or V (Figure 6), possibly owing to an increase in the forms of online working, online classes, and online entertainment (Nicola et al., 2020), leading to the conversion of some service electricity consumption into residential electricity consumption, which may have nothing to do with generating GDP. For example, home entertainment activities such as e‐games, the delay in the start of the school, and the cancellation or transfer of some cultural activities to online lead to lower EPC in recreation and shopping. Industry in Guangzhou was affected during the epidemic, but many biomedical companies continued to operate normally. Furthermore, many manufacturing companies in Guangzhou were diverted to the production of medical‐related goods after Chinese New Year (stage IV), and the process of resuming work and production accelerated in March, resulting in a relatively larger reduction in electricity consumption than that of Beijing but the smallest reduction in GDP among four cities. The epidemic disrupted human activities, which in turn affected energy consumption and economic development.

## 4 Discussion

### 4.1 Mechanisms Underlying the Effect of POIs on NTL Changes With the Development of the Epidemic

The combination of NTL radiance and POIs reveal more details of changes in human activities because of COVID‐19 and lockdown (Figures 5 and Figure 6). When the confirmed cases were first reported on 31 December 2019 in Wuhan, people did not realize the seriousness of COVID‐19, and production activities continued as usual. During Spring Festival (stage III), holiday population flows were still relatively high especially for the three cities of Beijing, Shanghai, and Guangzhou with lots of migrant workers. A large number of people returned to their hometown to spend the Chinese New Year with their families. Therefore, the declined contributions of the company and residence lead to the dimming of NTL at stage III. Since Wuhan was the city where the epidemic was first reported and the lockdown measures were first implemented, the population flow was first restrained leading to the increase of the residence contribution in Wuhan (Figure 6b). However, there is still a certain number of people who went out from Wuhan before lockdown leading. This population flow also caused COVID‐19 to spread to some other cities (Bai et al., 2020; Tian, Hu, et al., 2020) and led to a sharp rise in demand for medical‐related supplies such as medical masks. In response to the epidemic, many factories switched to manufacturing medical‐related products, thus the contribution of healthcare increased than case free period.

To prevent human‐to‐human transmission, cities have imposed strict lockdowns from the rising period (stage III), restricting people's movements and isolating them in their homes. Road closures, suspending intracity public transport, closure of recreational venues and service stations were implemented (Tian, Hu, et al., 2020). The direct result of stagnation in many fields is the declining contribution of company, recreation, and shopping. Additionally, festival celebrations were canceled or switched to online. These measures have led to a further reduction in NTL radiance. During the Spring Festival holiday, traffic, recreation, and shopping, which should be very active, showed a slump. During the outbreak period (stage IV), because of the COVID‐19 lockdown policy, many companies have implemented home working and online meetings, some extended holidays, and family entertainment increased (Nicola et al., 2020). Entering March (stage V), the epidemic gradually stabilized, the pace of resuming work and production gradually accelerated, the contribution of company and shopping increased and the contribution of residence decreased, with the corresponding NTL brightness recovering.

Lockdown measures reduced the spread of the epidemic, but they also reduced mobility and thus disrupted economic. As shown in Figure 6, even though the epidemic gradually stabilized in the stage V, the contribution of company did not return to the level of Stage I, indicating that the resumption of work and production was limited, rather than completely open. Therefore, post‐epidemic reopening strategies should be step‐by‐step. Meanwhile, the contribution of Shopping decreased after the epidemic began, indicating that lockdowns restricted mobility and economic activity in commercial areas. So preventing population concentrations, especially in company and commercial areas, is an effective control measure. But working at home and online shopping could be effective ways to reduce economic losses.

### 4.2 Limitations and Suggestions

We can see average R^2^ of RF models are only approximately 0.52 in this paper which is not very high. The reasons may include the following. (a) The types of light sources may be different (Elvidge et al., 2010; Wang et al., 2016). For example, the lights in residential areas are less bright than those in commercial areas. The general brightness of lighting in residential areas is relatively low, while the brightness of lighting in companies and businesses is high; (b) Limitations of POIs data: The traffic category only covers the corresponding facilities such as parking areas and bus stops, while the main light sources such as street lights and vehicle lights are not included. Furthermore, (c) The POIs data only captures the density of the six human activity categories, and the fact that several categories of lights are intermixed and interact with each other is not reflected in the POIs. For example, some shops are mixed with residential areas.

NTL images can be affected by air quality (Wang et al., 2016; Wang, Horby, et al., 2020). Air quality in China cities improved due to COVID‐19 Lockdown policy (Le et al., 2020; Su et al., 2020; Wang & Su, 2020). Although the VNP46A2 data is atmospherically calibrated, the calibration algorithm uses daytime‐to‐daytime averaged aerosol optical depth which may lead to errors in the NTL radiance. As alluded to earlier, the construction of new infrastructures such as the airport of Beijing (Figure 4) and the road of Wuhan (Figure S4 in Supporting Information S1) may have some influence on the analysis of the results. Besides, the satellite observation angles also affect NTL radiance (Li et al., 2019) which will be considered to develop adjustment methods in the future.

In our results, we proved that six categories of human activity indeed have an impact on changes in NTL radiance. But COVID‐19 also leads to changes in urban population flows which affects the NTL radiance. The contribution of each variable differs among the four cities in the five stages, mainly because lockdown changes lifestyle habits, and thus the detailed human activities need further investigation.

Previous studies have shown that there is a good correlation between GDP and NTL (Levin & Zhang, 2017; Ma et al., 2014), but this study suggests that the decline trends of GDP and NTL radiance are inconsistent among different cities which may be related to economically relevant changes in human activity during the epidemic. POIs may be further divided and analyzed by NTL for estimating GDP changes in different industries because NTL radiance corresponding to different POI changes differently at different stages.

## 5 Conclusions

Lockdown measures in China to stop the spread of COVID‐19 have significantly altered human activities and consequently socio‐economic. The specific manifestation of human activity in NTL was analyzed using POIs data, and the impact of the lockdown on GDP and EPC in four cities was analyzed. The main conclusions are mainly the following.

1. During COVID‐19 lockdown, the satellite observed NTL radiance declined in different levels from the confirmed cases appeared period. When the epidemic gradually stabilized, NTL began to recover but remained lower than the case free period.
2. Applying random forest, we found that approximately 52% of the variability in NTL was explained by the joint effect of six categories. Combining NTL radiance and POI, companies in all cities were resuscitated during stationary period, but Guangzhou's recovery was earlier. The traffic of Wuhan shows a different dynamic from other cities with a decline after city lockdown but some rebound during the outbreak period due to supports from other cities and new constructions. The rapid increase in shopping related NTL radiance in Wuhan also shows some recovery. Healthcare associated NTL radiance in Wuhan and Guangzhou increased rapidly when confirmed cases emerged, but declined somewhat after the outbreak of confirmed cases.
3. The variation trend of GDP (or EPC) is in line with NTL radiance changes. But the decline of GDP is not exactly corresponding to the decline in NTL radiance, which is related to the specific details of the resumption of work and production in different cities.

On the whole, we conclude that the lockdown policy plays an important role in the war against COVID‐19 in China, and the effect of lockdown measures on human activity and consequently on the socio‐economic can be mapped by satellite NTL. The above findings may be useful for the development of more effective control measures, post‐epidemic reopening strategies, outbreak control in the worldwide COVID‐19 fighting, or other infectious disease control.

## Conflict of Interest

The authors declare no conflicts of interest relevant to this study.

## Supporting information

Supporting Information S1

## Data Availability

Data sets for this study are publicly available online. Visible Infrared Imaging spectroRadiometer Suite nighttime light data VNP46A2 can be found online at https://ladsweb.modaps.eosdis.nasa.gov/. Points of interest data in 2020 of four cities can be obtained from Baidu Map, and are available from https://github.com/wangunji/POI‐data‐of‐China/, only available via GitHub. Coronavirus disease 2019 epidemic cases data are available at https://github.com/Estelle0217/COVID‐19‐Epidemic‐Dataset, only available via GitHub. Gross domestic product data and electric power consumption data are collected from the National Bureau of Statistics and local statistical bureaus, and they are publicly available from https://github.com/wangunji/social‐economic‐data, only available via GitHub. Land use data in 2018 of four cities can be found from http://www.resdc.cn/Default.aspx.
