# Supplementary material for: Human Activity Changes During COVID‐19 Lockdown in China—A View From Nighttime Light
Source: Geohealth. 2022 Aug 1;6(8):e2021GH000555. doi: 10.1029/2021GH000555 (PMC9350096; doi:10.1029/2021GH000555)
Supplement: Supplementary file 1 — Supporting Information S1 [file GH2-6-e2021GH000555-s001.docx]

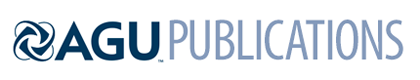


*GeoHealth*

Supporting Information for

**Human Activity Changes during COVID-19 Lockdown in China—A View of Nighttime Light**

Xuejun Wang^1, 2^, Guangjian Yan^1, 2, †^, Xihan Mu^1, 2^, Donghui Xie^1, 2^, Jiachen Xu^1, 2^, Zhiyu Zhang^1, 2^, and Dingdan Zhang^1, 2^

^1^State Key Laboratory of Remote Sensing Science, Jointly Sponsored by Beijing Normal University and Aerospace Information Research Institute, Chinese Academy of Sciences, Beijing, China, ^2^Beijing Engineering Research Center for Global Land Remote Sensing Products, Faculty of Geographical Science, Beijing Normal University, Beijing, China ^†^Corresponding Author

**Contents of this file**

Text S1

Figures S1 to S7

Table S1 to S4

**Introduction**

Text S1 provides further details on the processing of the nightlight remote sensing data. Figures S1-S7 are cited in the main body of the paper to provide extra evidence to support the analysis. Table S1-S4 is referenced in the paper to provide detailed information of the analysis.

Text S1. Data preprocessing

VIIRS/NPP Gap-Filled Lunar BRDF-Adjusted Nighttime Lights Daily L3 Global Linear Lat Lon Grid (VNP46A2) products were preprocessed as follows.

1. Step 1: The nighttime light (NTL) radiance data and quality flags (QF) in the dataset were extracted, and merged to obtain full images of the four cities.
2. Step 2: NTL data were checked using QF, and only high quality pixels were reserved. In addition, high energy particles with radiance larger than 1000 nW·m^-2^sr^-1^ were discarded ([Elvidge et al., 2017](#_ENREF_1)).
3. Step 3: According to the boxplot metrics, outliers were removed. More specifically, the 25th percentile (Q1) and 75th percentile (Q3) were firstly calculated, which in turn yielded the interquartile range (IQR=Q3-Q1). Observations outside the Q1 -1.5*IQR and Q3 + 1.5*IQR ranges were considered outliers and were excluded from the NTL data ([Román et al., 2021](#_ENREF_2)).
4. Step 4: The NTL data were divided into five stages and all images in each stage were averaged (The date of the Lunar New Year varies every year, so the corresponding time division varied each year, as shown in Table S2).
5. Step 5: The unnatural landscape areas of NTL images were extracted for each stage in different years, and the average NTL radiance of these regions in different cities were calculated.


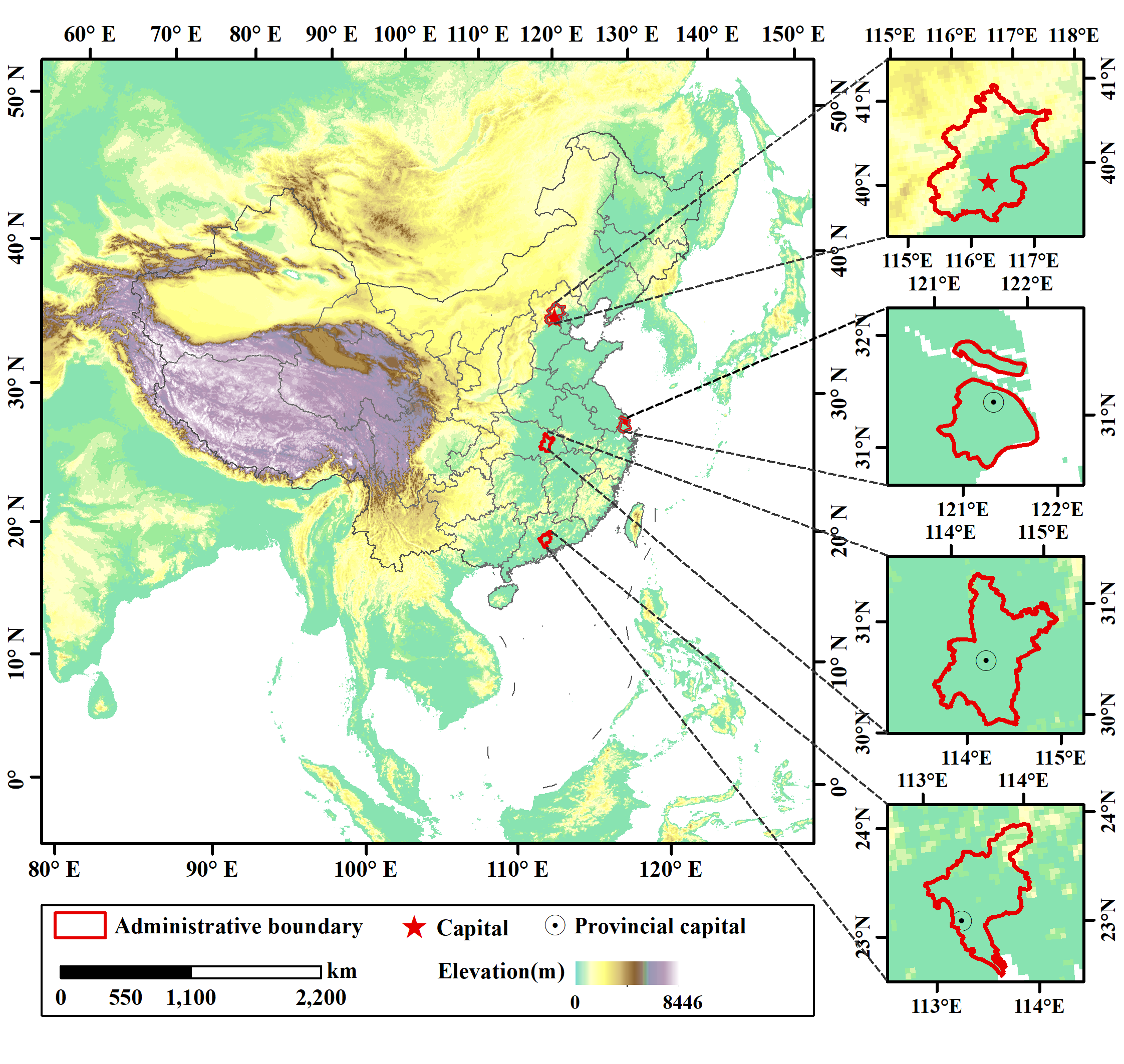


Figure S1. Study areas.


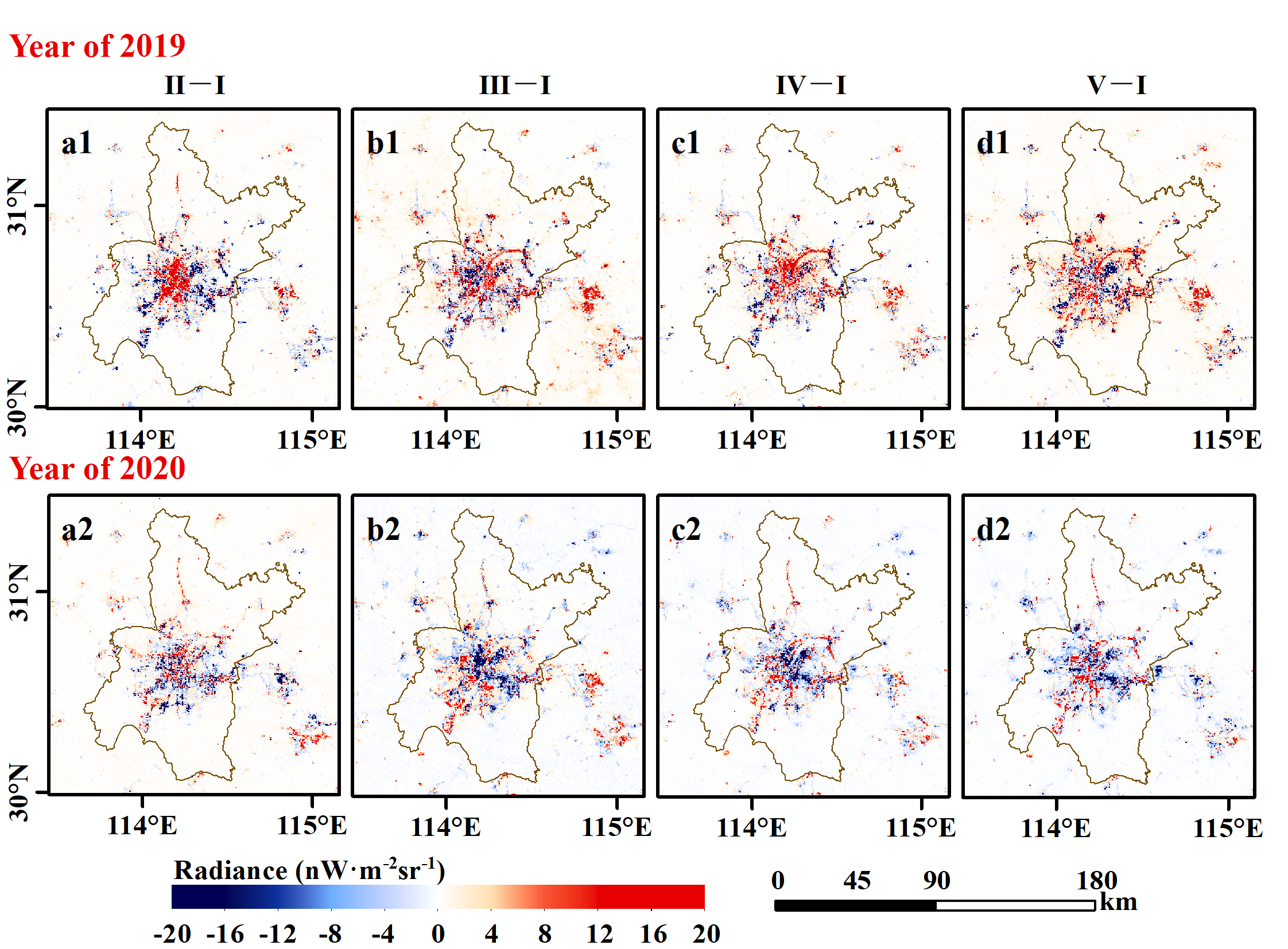


Figure S2. NTL radiance changes in COVID-19 development stages of Beijing. Upper panel and bottom panel depict the NTL radiance change in 2019 and 2020, respectively. The columns from left to right are NTL radiance differences (a) between stage II and stage Ⅰ, (b) between stage Ⅲ and stage Ⅰ, (c) between stage Ⅳ and stage Ⅰ, and (d) between stage Ⅴ and stage Ⅰ. Compared to the NTL radiance in 2019, the white part indicates little or no NTL change, the red part indicates brightening and the blue part indicates dimming.


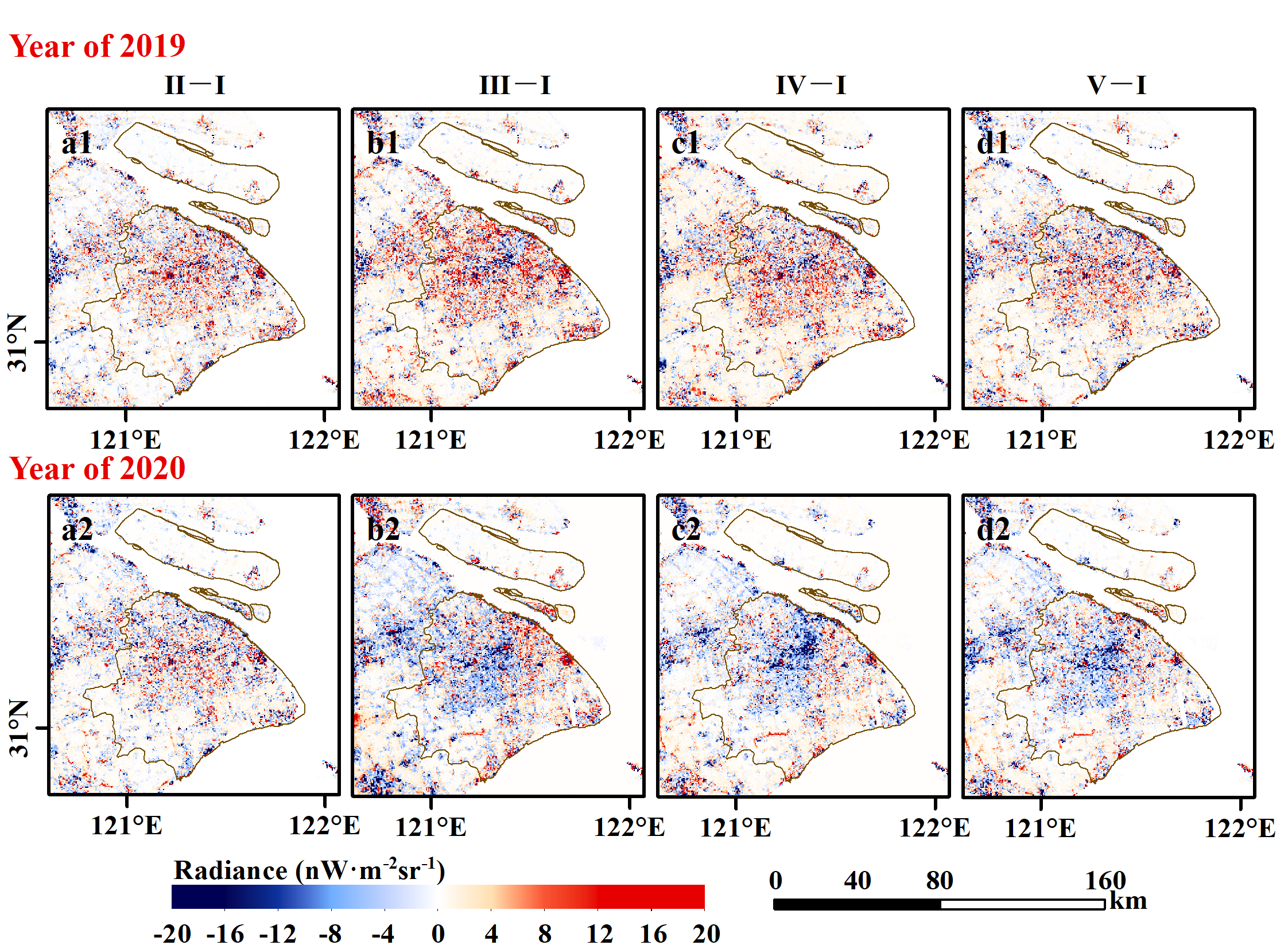


Figure S3. Same as Figure S1 but for Shanghai.


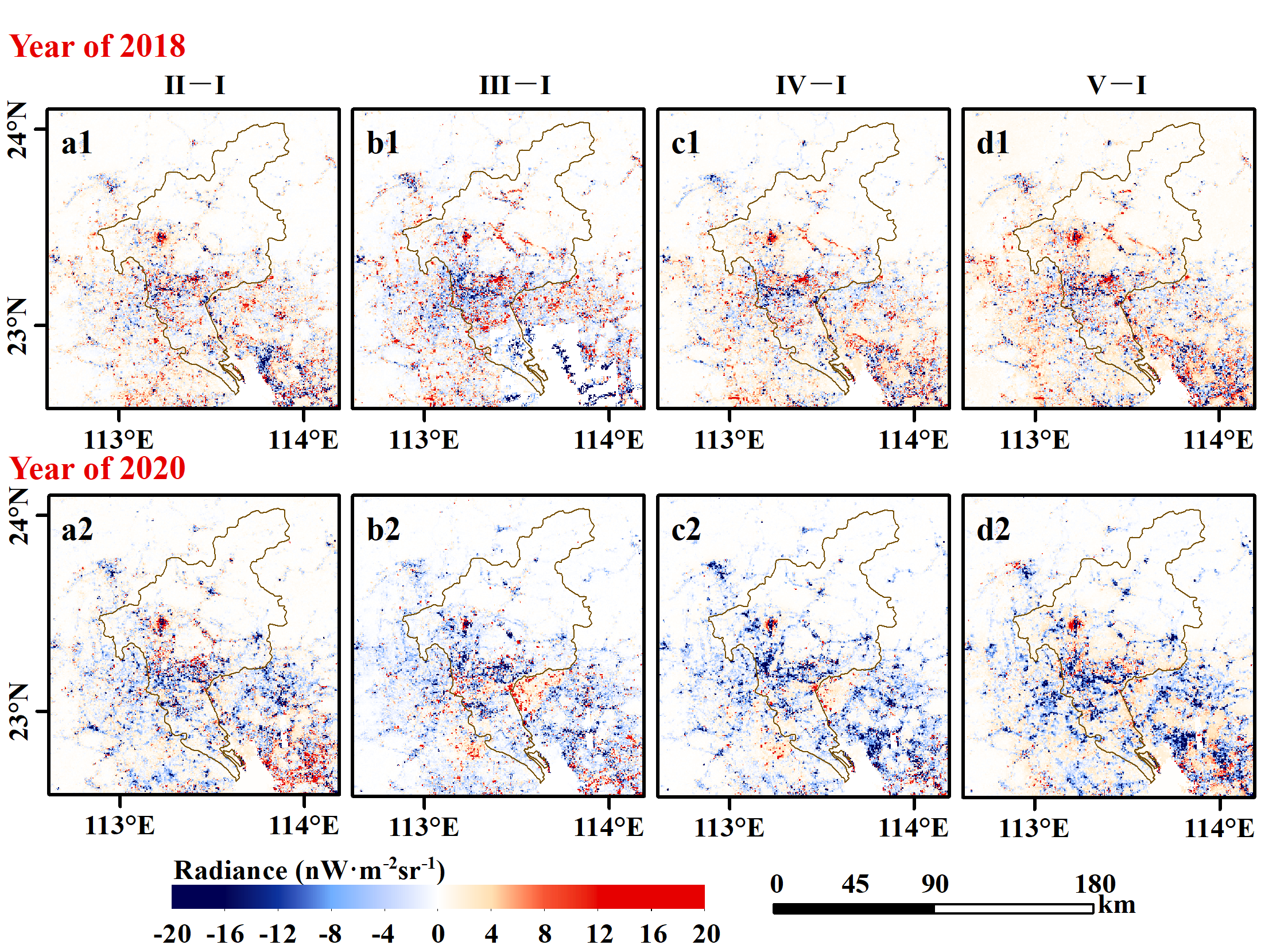


Figure S4. Same as Figure S1 but for Guangzhou. Upper panel depicts the NTL radiance change in 2018 and the bottom panel depicts the NTL radiance change in 2020 (Due to some missing data in stage Ⅲ and Ⅴ of 2019, the results are compared with those of 2018).


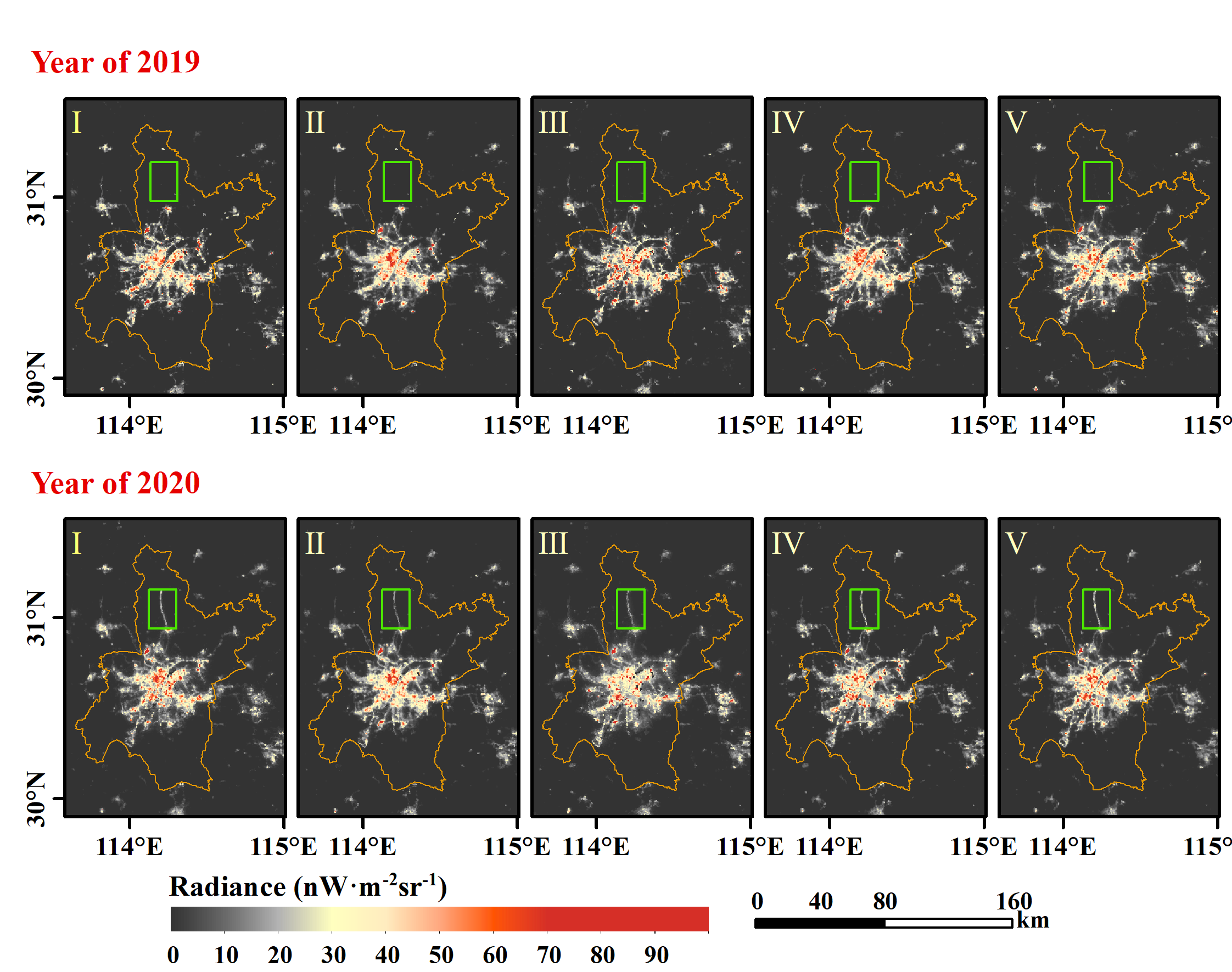


Figure S5. Average NTL radiance distribution of Wuhan in different stages. The upper panel depicts the NTL radiance in 2019 and the bottom panel depicts the NTL radiance in 2020. Regions in the green rectangles mark the Mulan Avenue in Wuhan, which reveal the brightening of the NTL in 2020 compared to 2019.


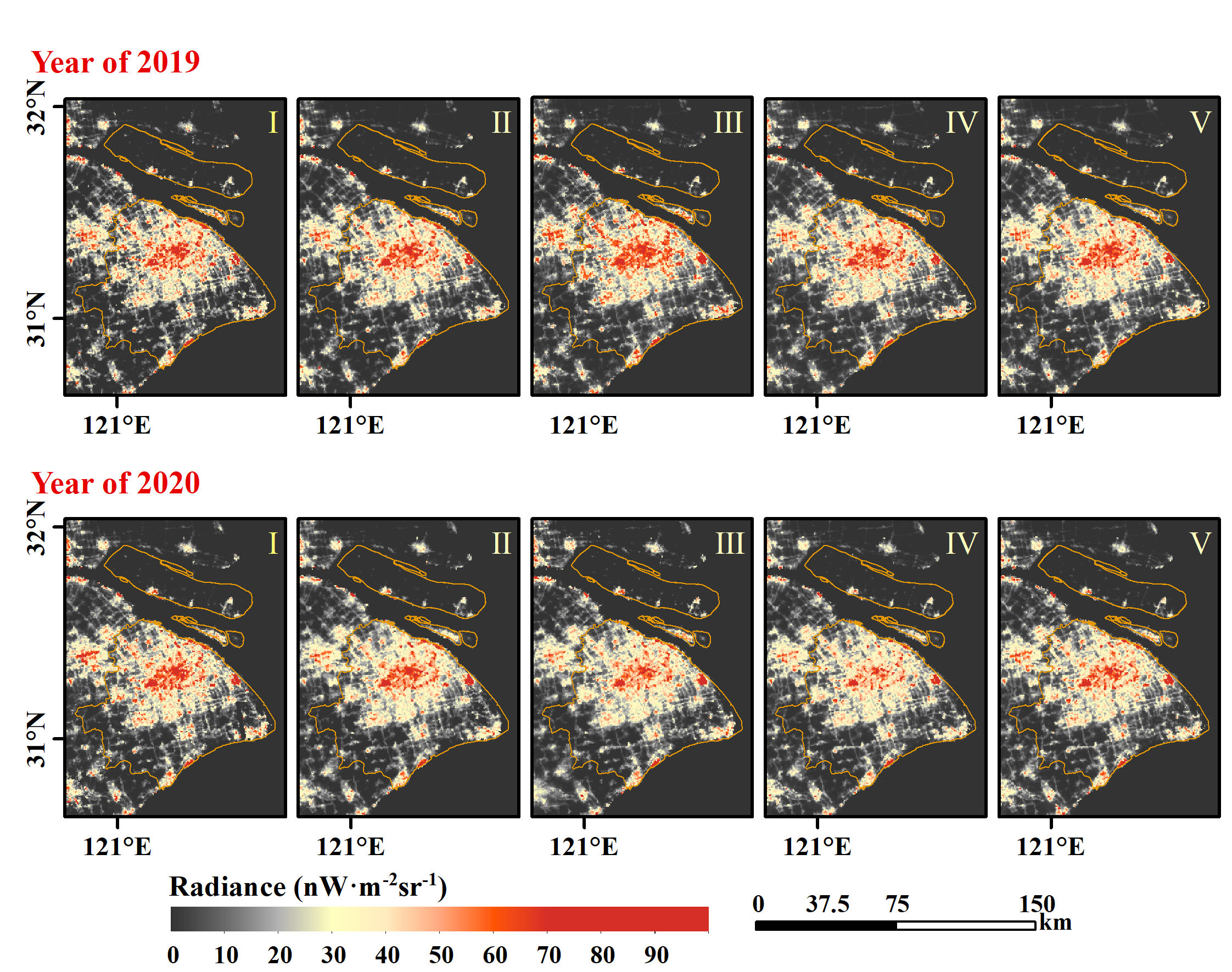


Figure S6. Same as Figure S4 but for Shanghai.

**
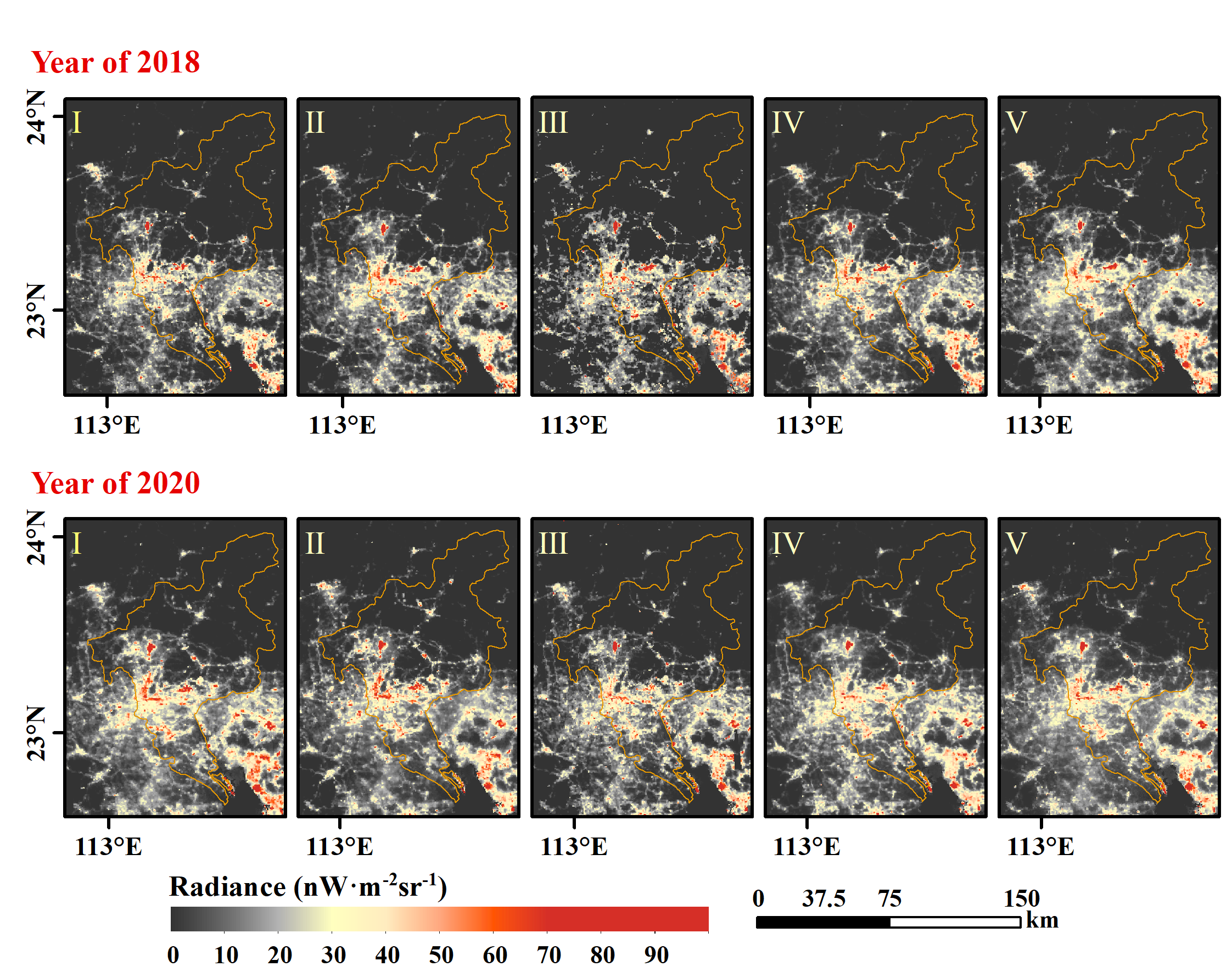
**

Figure S7. Average NTL radiance distribution of Guangzhou in different stages. The upper panel depicts the NTL radiance in 2018 and the bottom panel depicts the NTL radiance in 2020.

| City | Land areas (km^2^)^b^ | Unnatural landscape areas (%)^a^ | Permanent residents (×10^4^)^c^ | Total confirmed cases at 31 March 2020 |
| --- | --- | --- | --- | --- |
| Beijing | 16410.5 | 21.30 | 2153.6 | 580 |
| Wuhan | 8569.2 | 16.03 | 1121.2 | 50007 |
| Shanghai | 6340.5 | 45.17 | 2428.1 | 516 |
| Guangzhou | 7434.4 | 19.97 | 1530.6 | 440 |

*Note.* ^a^The unnatural landscape areas (Urban and rural, industrial and mining, residential land) of four cities were obtained using land use data. ^b^Land areas and ^c^permanent residents are obtained from Statistical Yearbook for 2019 of four cities.

Table S1. Basic information of the four cities

| Stage | 2016 | 2017 | 2018 | 2019 | 2020 |
| --- | --- | --- | --- | --- | --- |
| Ⅰ | 15-31 December 2015 | 15-31 December 2016 | 15-31 December 2017 | 15-31 December 2018 | 15-31 December 2019 |
| Ⅱ | 1 January to 4 February | 1-24 January | 1 January to 12 February | 1 January to 1 February | 1-21 January |
| Ⅲ | 5-15 February | 25 January to 4 February | 13-23 February | 2-12 February | 22 January to 1 February |
| Ⅳ | 16 February to 14 March | 5 February to 4 March | 24 February to 23 March | 13 February to 12 March | 2-29 February |
| Ⅴ | 15-31 March | 5-31 March | 24-31 March | 13-31 March | 1-31 March |

*Note.* Data of stage Ⅰ (reference) are all from the previous year.

Table S2. Stage divisions in each year.

| City | Company | Healthcare | Recreation | Residence | Shopping | Traffic facility |
| --- | --- | --- | --- | --- | --- | --- |
| Beijing | 123042 | 15425 | 19909 | 174385 | 81117 | 13240 |
| Wuhan | 51556 | 7126 | 9722 | 112030 | 59663 | 9989 |
| Shanghai | 197921 | 11137 | 17512 | 259596 | 92960 | 23089 |
| Guangzhou | 136505 | 10442 | 11589 | 45911 | 100859 | 11014 |

Table S3. Number of POIs in four cities

| City | Beijing | Wuhan | Shanghai | Guangzhou |
| --- | --- | --- | --- | --- |
| NTL radiance (nW·m^-2^sr^-1^) | -0.32 | -0.58 | -0.72 | -0.30 |
| GDP (Billion RMB) | -3.22 | -11.00 | -5.40 | -2.79 |
| EPC (TWh) | -1.04 | -3.48 | -4.59 | -1.92 |
| R^2^ (GDP and NTL) | 0.36 | | | |
| R^2^ (EPC and NTL) | 0.92 | | | |

Table S4. Decline details between Q1 of 2020 and 2019. The reduction values of NTL radiance, GDP, and EPC compared to Q1 of 2019 are calculated, and R^2^ values were further derived (NTL radiance and GDP, NTL radiance, and EPC).

References

Elvidge, C. D., Baugh, K., Zhizhin, M., Hsu, F. C., &Ghosh, T. (2017), VIIRS night-time lights, *International Journal of Remote Sensing*, *38*(21), 5860-5879. <http://dx.doi.org/10.1080/01431161.2017.1342050>

Román, M. O., Wang, Z., Shrestha, R., Yao, T., &Kalb, V. (2021), *Black marble user guide version 1.2*, Washington, DC: National Aeronautics and Space Administration.
